# Eed-dependent histone modification orchestrates the iNKT cell developmental program alleviating liver injury

**DOI:** 10.3389/fimmu.2024.1467774

**Published:** 2024-09-20

**Authors:** Yun Guo, Shun Ohki, Yohei Kawano, Weng Sheng Kong, Yoshinori Ohno, Hiroaki Honda, Masamoto Kanno, Tomoharu Yasuda

**Affiliations:** ^1^ Department of Immunology, Graduate School of Biomedical and Health Sciences, Hiroshima University, Hiroshima, Japan; ^2^ Department of Biochemistry, Faculty of Medicine, Fukuoka University, Fukuoka, Japan; ^3^ Field of Human Disease Models, Major in Advanced Life Sciences and Medicine, Tokyo Women’s Medical University, Tokyo, Japan; ^4^ Medical Research and Development Programs Focused on Technology Transfers: Development of Advanced Measurement and Analysis Systems (AMED-SENTAN), Japan Agency for Medical Research and Development, Tokyo, Japan; ^5^ Japan Agency for Medical Research and Development-Core Research for Evolutionary Medical Science and Technology (AMED-CREST), Japan Agency for Medical Research and Development, Tokyo, Japan

**Keywords:** iNKT, Eed, H3K27me3, PRC2, liver injury

## Abstract

Polycomb repressive complex 2 (PRC2) is an evolutionarily conserved epigenetic modifier responsible for tri-methylation of lysine 27 on histone H3 (H3K27me3). Previous studies have linked PRC2 to invariant natural killer T (iNKT) cell development, but its physiological and precise role remained unclear. To address this, we conditionally deleted Eed, a core subunit of PRC2, in mouse T cells. The results showed that Eed-deficient mice exhibited a severe reduction in iNKT cell numbers, particularly NKT1 and NKT17 cells, while conventional T cells and NKT2 cells remained intact. Deletion of Eed disrupted iNKT cell differentiation, leading to increased cell death, which was accompanied by a severe reduction in H3K27me3 levels and abnormal expression of *Zbtb16*, *Cdkn2a*, and *Cdkn1a*. Interestingly, Eed-deficient mice were highly susceptible to acetaminophen-induced liver injury and inflammation in an iNKT cell-dependent manner, highlighting the critical role of Eed-mediated H3K27me3 marks in liver-resident iNKT cells. These findings provide further insight into the epigenetic orchestration of iNKT cell-specific transcriptional programs.

## 1 Introduction

The functional repertoire of T cells is shaped by highly ordered development and selection processes in the thymus, driven by T-cell receptor (TCR) rearrangement and signal strength, which enables the thymus to contribute to host immunity (1–3). Immature CD4/CD8 double-positive (DP) progenitors expressing distinct αβ TCRs undergo positive selection in the thymus. Those with lower-affinity TCRs are selected for weak reactivity to MHC, eventually becoming naive CD4^+^ and CD8^+^ conventional T cells, while agonist-selected those with higher TCR affinity become regulatory T cells (Tregs) or invariant natural killer T cells (iNKTs) (4, 5). Although Tregs continue to perceive higher TCR signals in the periphery, iNKT cells stop receiving TCR signals as they mature and migrate (6).

iNKT cells, an innate-like unconventional T-cell subset, exhibit unique effector and memory phenotypes, promptly secrete multiple cytokines upon activation (IFN-γ, GM-CSF, IL-2, IL-17, and TNF-α), and are involved in various diseases, including inflammatory conditions (7–9). The invariant TCRs (Vα14-Jα18 in mice; Vα24-Jα18 in humans) expressed in iNKT cells recognize lipid antigens presented by the MHC class I-like molecule CD1d on other DP thymocytes, along with SLAM family receptor co-stimulation (7, 10–12). Upon commitment to the iNKT cell lineage, they undergo sequential differentiation stages: S0 (CD24^+^CD44^-^NK1.1^-^), S1 (CD24^-^CD44^-^NK1.1^-^), S2 (CD24^-^CD44^+^NK1.1^-^), and S3 (CD24^-^CD44^+^NK1.1^+^) mature iNKT cells (7, 13). PLZF, encoded by *Zbtb16*, is induced by TCR signaling and is required for the development and functionality of iNKT cells (14, 15). Expressions of T-bet, GATA3, or RORγt transcription factors instruct NKT progenitors (NKTp) to IFN-γ-secreting NKT1, IL-4-secreting NKT2, or IL-17-secreting NKT17 effector cells, respectively (16).

Cell lineage commitment and specification rely on coordinated gene expression and epigenetic regulation (17). Histone H3 lysine 27 trimethylation (H3K27me3), associated with transcriptional repression, is facilitated by the enzymatic activity of polycomb repressive complex 2 (PRC2), comprising Eed, Suz12, and Ezh1/Ezh2 subunits and maintains specific gene expression patterns during development. Ezh1 and Ezh2 are catalytic subunits of the PRC2 methyltransferase for H3K27me3, which requires Eed and Suz12 for catalytic activation (18, 19). Eed recognizes H3K27me3, inducing a conformational change in PRC2 and activating the Ezh2 enzyme. The proposed positive feedback loop model involves initial H3K27me3 deposition by PRC2. Subsequently, PRC2 is further recruited through the binding of its Eed subunit to H3K27me3, which allosterically activates PRC2. This leads to additional H3K27me3 deposition, enabling stable chromatin domains (20, 21). Thus, Eed is essential for both initial activation and amplifying PRC2 activity for H3K27me3. Deficiency of Ezh2 in mouse T cells partially reduces H3K27me3 levels in iNKT cells and accumulates Ezh2-deficient iNKT cells, suggesting that incomplete loss of PRC2 activity perturbs iNKT cell development (22, 23). Conversely, removing Eed or Suz12 from T cells severely inhibits iNKT cell development, highlighting the essential role of PRC2 activity (23). However, the underlying epigenetic mechanisms directing iNKT cells from DP thymocytes and those required for iNKT effector subset differentiation remain unknown. Therefore, we conditionally deleted Eed from DP thymocytes in mice to investigate its role in iNKT cell lineage commitment and effector subset differentiation. We further analyzed the physiological effect of Eed-dependent iNKT cells on liver homeostasis in response to hepatocyte death and associated inflammation.

## 2 Materials and methods

### 2.1 Mice

Eed-flox mice were backcrossed to C57BL/6 for more than 7 generations (24). CD4-Cre transgenic mice were crossed with Eed-flox mice to generate Eed conditional knockout (cKO) mice (25). C57BL/6 (Ly5.2) mice were obtained from CLEA Japan and C57BL/6 background Ly5.1 mice were obtained from the Jackson Laboratory. Mice were bred and maintained under the specific pathogen-free conditions.

### 2.2 Flow cytometry

Single-cell suspensions from the spleen and bone marrow were resuspended in Gey’s or ACK solutions for red blood cell lysis. Cells were treated with Fc Block (2.4G2, BD Biosciences) or TruStain FcX (93, BioLegend) followed by staining with fluorochrome-conjugated antibodies or biotinylated antibodies. Anti-CD24 (M1/69), CD44 (IM7), TCRβ (H57-597), NK1.1 (PK136), CD4 (RM4-5), CD8 (53-6.7), PLZF (R17-809), RORγt (Q31-378), Ly49A (JR9-318), Ly49C and Ly49I (5E6), Ly49G2 (4D11), IL-4 (11B11), IL-17A (TC11-18H10), IFN-γ (XMG1.2) were purchased from BD Biosciences; Anti-T-bet (4B10) was purchased from BioLegend. The cells stained with biotinylated antibodies were detected by fluorochrome-conjugated streptavidin. Dead Cells were excluded using propidium iodide (Sigma-Aldrich). To detect iNKT cells, the cells were labeled with α-GalCer (Funakoshi)-loaded CD1d-tetramer PE (MBL) for 30 min at room temperature. For intracellular staining, the cells were fixed and permeabilized using a Foxp3 staining kit (Thermo Fisher Scientific). For cytokine staining, the cells were treated with 50 ng/ml of phorbol 12-myristate 13-acetate (PMA) and 1 μg/ml of ionomycin with BD GolgiStop containing monensin for 6 hours (h) followed by staining using Cytofix/Cytoperm Plus Fixation/Permeabilization Kit (BD Biosciences). For H3K27me3 and Eed staining, anti-trimethyl-Histone H3 (Lys27) polyclonal antibody (07-449, Upstate), anti-Eed (E4L5E) XP rabbit monoclonal antibody (85322, CST), and normal rabbit IgG (2729; CST) as isotype control were labeled using Zenon Alexa Fluor 647 rabbit IgG Labeling Kit (Z25308; Invitrogen) followed by intracellular staining procedure. Stained cells were analyzed using FACSCanto II (BD Biosciences) or CytoFLEX S (Beckman Coulter). Cell sorting was performed using FACSAria II (BD Biosciences). Data were analyzed on FlowJo software (BD Biosciences).

### 2.3 Isolation of lymphocytes from the liver and lung

To isolate lymphocytes from the liver and lung, mice were anesthetized and perfused with PBS. For liver lymphocytes, the liver cells were filtered through 100-μm nylon mesh, washed with 2% FBS/PBS, and resuspended in 33.75% Percoll PLUS (GE Healthcare Life Sciences) followed by centrifugation at 2,300 rpm for 25 min at room temperature. For lung lymphocytes, the lung was cut into small pieces and digested with 1 mg/ml collagenase D (Roche) and 0.5 mg/ml DNase I (Roche) for 1 h at 37°C with shaking. The cells were filtered through 70-μm nylon mesh and then treated with Gey’s solution to remove red blood cells.

### 2.4 Fetal thymus organ culture

Fetal thymus organ culture (FTOC) was performed as described previously (26). For timed pregnancies, the day of the vaginal plug was designated E0.5. The thymic lobes were isolated from control or Eed cKO E15.5 fetuses, which were then transferred to standard FTOC conditions and analyzed for iNKT cell development on the indicated day.

### 2.5 Generation of BM mixed chimeras

Bone marrow (BM) cells (2x10^6^) obtained from control or Eed cKO mice (Ly5.2) were mixed with C57BL/6 (Ly5.1/5.2) BM cells at a 1:1 ratio and transferred into 9-Gy irradiated C57BL/6 (Ly5.1) recipient mice. Recipient mice were analyzed after 4 months (Mon).

### 2.6 Acetaminophen (N-acetyl-para-aminophenol, APAP)-induced liver injury

APAP (00204-82, Nacalai Tesque) was dissolved at 15 mg/ml in PBS at 37°C. Seven- to eleven-week-old control and Eed cKO mice were fasted for 16 h before APAP treatment. For liver histology, female mice were administrated with 360 mg/kg APAP and sacrificed mice at 48 or 72 h after treatment. Hematoxylin and eosin (H&E) staining was performed with frozen liver tissues, firstly stained with Mayer’s Hematoxylin Solution (FUJIFILM) and 1% Eosin Y Solution (FUJIFILM), then followed by ethanol and xylene dehydration steps. The images were obtained from BZ-X800 microscope (Keyence). The percentage of necrosis area was measured by Keyence analysis software (Keyence). For the inflammation score (27), severity was categorized into four levels: 0, no inflammation; 1, moderate and inflammatory cells were scattered; 2, marked and inflammatory cells formed foci; 3, severe and cells were inflammatory diffuse. For iNKT transfer experiments, liver iNKT cells sorted from wild-type Ly5.1 mice were injected intravenously into control or Eed cKO mice at 3-5x10^5^ cells per mouse, then fasted mice were treated with APAP after 3 days. To analyze the survival, male mice were injected 375 mg/kg APAP by intraperitoneal and checked mice survival every 12 h.

### 2.7 RNA-seq

CD4^+^CD8^+^TCRβ^+^CD69^+^ thymocytes (1x10^5^) were sorted from 3 pairs of each control or Eed cKO mice, then kept in RNA later solution (Thermo Fisher Scientific). Total RNA was isolated by using QIAzol and miRNeasy Micro kit (QIAGEN). cDNA synthesis (1x105) was used SMART-Seq v4 Ultra Low Input RNA Kit for Sequencing Kit (Takara). Libraries were prepared by Nextera XT v2 Kit (Illumina) and sequenced on an Illumina HiSeq 2500. Quality control and adapter trimming for raw reads were performed by fastp (v0.21.0). Trimmed read mapping and quantification were performed with RSEM (v1.3.1) using STAR (v2.7.10) as an aligner. Differential expressions were identified by using DEseq2 (v1.34.0). RNA-seq data were used for GSEA analysis with gene sets obtained from publicly available databases (28, 29).

### 2.8 ChIP-seq

H3K27me3 ChIP-seq dataset was obtained from GSE84238 (30). ChIP-seq raw reads were mapped onto reference mouse genome mm39 by using HISAT2 and peaks were called using MACS2. SparK and IGV were used to visualize bedGraph coverage tracks.

### 2.9 ChIP-qPCR

DP and iNKT cells sorted from control and Eed cKO mice were fixed with 1% formaldehyde for 10 min at room temperature and sonicated with Bioruptor, and then sonicated chromatins were immunoprecipitated by control rabbit IgG antibody (2729, CST) or anti-trimethylated H3K27 antibody (07-449, Upstate) coupled with SureBeads Protein A/G Magnet Beads (Bio-Rad) overnight. After washing and reverse crosslinking, DNA was purified using Phenol: Chloroform: Isoamyl alcohol (Nacalai Tesque). qPCR was performed using Thunderbird SYBR qPCR mix (TOYOBO) or TB Green Premix Ex Taq II (Takara). Primers are listed in **Supplementary Table 1**.

### 2.10 Statistical analysis

All data analyses were performed with Prism 7 software (GraphPad). The results were presented as mean ± SD. *p* values were calculated by unpaired t-test, multiple t-tests with the Holm-Sidak method at α = 0.05, or one-way ANOVA. Statistical significance was determined with alpha<0.05 and presented as *, <0.05; **, <0.01; ***, <0.001; ****, <0.0001.

## 3 Results

### 3.1 Eed is essential for iNKT cell development

**Figure 1:**
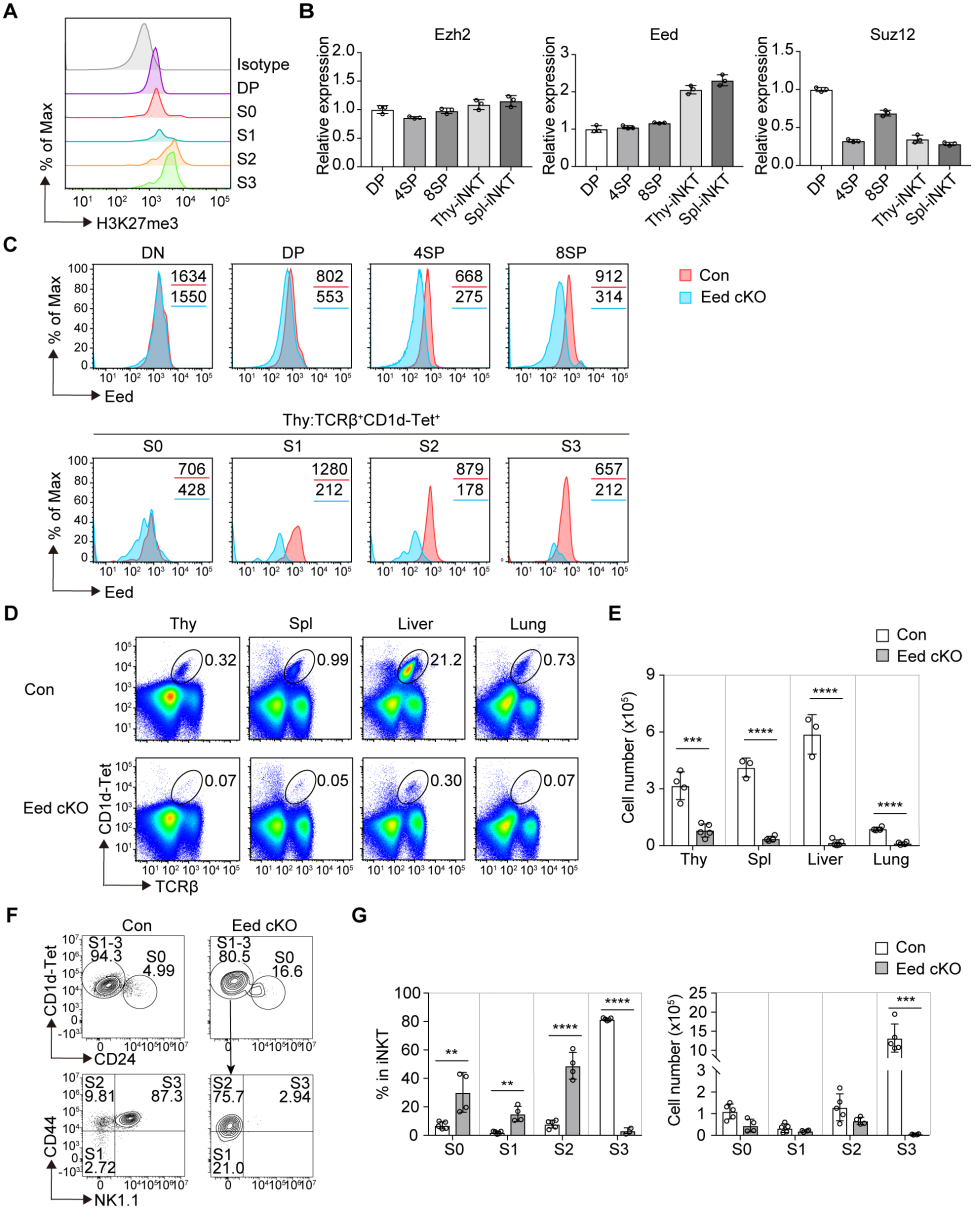
Eed is essential for iNKT cell development. **(A)** FACS analysis of H3K27me3 levels in the indicated thymic iNKT cell populations. The gray histogram indicates isotype control. S0, CD24^+^; S1, CD24^-^CD44^-^NK1.1^-^; S2, CD24^-^CD44^+^NK1.1^-^; and S3, CD24^-^CD44^+^NK1.1^+^ cells are gated on TCRβ^+^CD1d-Tet^+^. DP, CD4^+^CD8^+^. **(B)** Real-time PCR analysis of the expression of the PRC2 core members Ezh2, Eed, and Suz12 in the indicated T-cell subsets. Expression values were normalized to *Ywhaz*. DP, CD4^+^CD8^+^; 4SP, CD4^+^CD8^-^; 8SP, CD4^-^CD8^+^ from thymus. iNKT, TCRβ^+^CD1d-Tet^+^cells from thymus (Thy) and spleen (Spl). **(C)** Eed expression in the indicated cell populations of control (*red*) and Eed cKO (*blue*) mice. The values in each panel show the mean fluorescence intensity (MFI). DN, CD4^-^CD8^-^; DP, CD4^+^CD8^+^; 4SP, CD4^+^CD8^-^; and 8SP, CD4^-^CD8^+^ cells from thymus. **(D, E)** Representative FACS plots and absolute number of TCRβ^+^CD1d-Tet^+^ iNKT cells in the thymus, spleen, liver, and lung from control (n=3-4) and Eed cKO mice (n=4-5). **(F)** Representative FACS plots of the thymic iNKT populations from control and Eed cKO mice. Expression of CD44 and NK1.1 are analyzed on CD1d-Tet^+^CD24^-^ fraction corresponding to S1-3 stages of iNKT cells. **(G)** Percentage and absolute cell number of the indicated cell populations from **(F)** of control (n=5) and Eed cKO mice (n=4). Data are mean ± SD with statistical significance determined by unpaired t-tests **(E)** or multiple t-test **(G)**. *p* values are represented as **, <0.01; ***, <0.001; ****, <0.0001. Data are representative of at least two independent experiments.

We examined H3K27me3 levels during iNKT cell development. H3K27me3 signals were detected in developing thymic iNKT cells from DP to the S3 stage, indicating that H3K27me3 histone modification occurs during the early development of iNKT cells. Notably, H3K27me3 levels significantly increased during the transition from DP/S0 to the S2 stage, suggesting that additional activation of the PRC2 complex leads to *de novo* deposition of H3K27me3 with stage progression (**Figure 1A**). We compared the mRNA expression of PRC2 core subunits among the T-cell subsets. Similar to the H3K27me3 levels, *Eed* mRNA expression levels were increased in thymic and splenic iNKT cells compared to thymic DP cells, whereas *Ezh2* and *Suz12* mRNAs were equal or downregulated (**Figure 1B**). These results prompted us to investigate the specific function of Eed in iNKT cell development.

We bred Eed-flox mice with CD4-Cre transgenic mice expressing Cre recombinase after the DP stage in the T-cell lineage (named Eed cKO mice). We first examined the deletion of Eed in different T-cell populations in Eed cKO mice. The expression of Eed protein remained intact in double-negative (DN) cells but was partially abrogated in DP cells and S0 iNKT cells, with nearly complete loss of Eed in CD4 or CD8 single-positive (SP) cells, S1/3 iNKT cells, and splenic T cells (**Figure 1C**; **Supplementary Figure 1A**). Additionally, *Eed* mRNA was severely decreased in the S1 to S3 stages, but only partially decreased in the S0 stages of iNKT cells from Eed cKO mice (**Supplementary Figure 1B**). These results confirmed Eed protein expression in conventional T cells and iNKT cells, consistent with the mRNA expression profile, indicating that the deletion of Eed using CD4-Cre resulted in a delay beyond the thymic DP cell stage, similar to previous findings (31).

In Eed cKO mice, we observed a significant reduction in iNKT cells characterized by TCRβ^+^CD1d-tetramer (Tet)^+^ cells in various tissues, including the thymus, spleen, liver, and lung (**Figure 1D, E**). To further examine the early developmental stages of impaired thymic iNKT cells in Eed cKO mice, we utilized the surface markers CD24, CD44, and NK1.1, to distinguish the developmental stages of iNKT cells. Compared to control mice, Eed cKO mice showed a significant increase in the percentage of iNKT cells from the S0 to S2 stage, followed by a substantial decline at the S3 stage (**Figures 1F, G**). Furthermore, the absolute number of S3 iNKT cells in Eed cKO mice was severely reduced compared to that of control mice, indicating a critical role of Eed in the S2 to S3 transition during iNKT cell development, and the increased proportions of S0, S1, and S2 iNKT cells were mainly due to the reduction of the S3 iNKT cells. Conversely, the numbers of DN, DP, CD4 SP, CD8 SP, and Foxp3^+^ Tregs T-cell subsets were not affected by Eed deficiency in thymus (**Supplementary Figure 2**). These results suggest that Eed plays an indispensable and specific role in the development of iNKT cells.

### 3.2 Age-dependent and cell-intrinsic role of Eed in iNKT cell development

**Figure 2:**
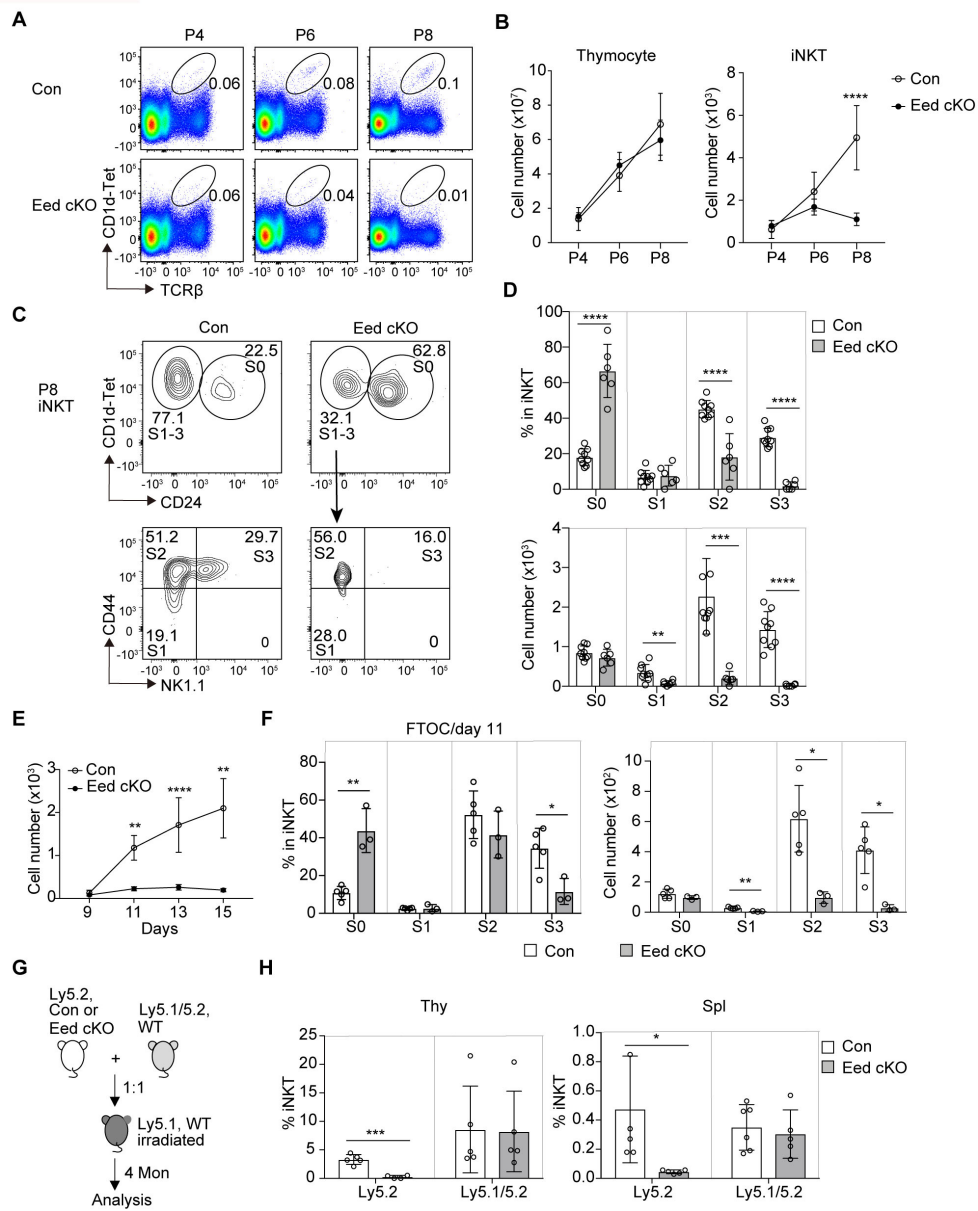
Age-dependent and cell-intrinsic role of Eed in the development of iNKT cells. **(A)** Representative FACS plots of TCRβ^+^CD1d-Tet^+^ iNKT cells in the thymus of control and Eed cKO mice at postnatal day 4 (P4), 6 (P6), and 8 (P8). **(B)** Absolute numbers of total thymocytes and TCRβ^+^CD1d-Tet^+^ iNKT cells in the thymus of control and Eed cKO mice at P4 (n>3), P6 (n>5), and P8 (n>5). **(C, D)** Representative FACS plots, percentage, and absolute cell number of the indicated thymic iNKT populations from control and Eed cKO mice at P8. **(E, F)** E15.5 fetal thymic lobes were isolated from control or Eed cKO mice and cultured on FTOC condition. **(E)** Absolute number of TCRβ^+^CD1d-Tet^+^ iNKT cells in control and Eed cKO fetal thymus on days 9, 11, 13, and 15 of culture. The number of lobes in FTOC cultures: day 9 (n=3-4), day 11 (n=3-5), day 13 (n=6-10), and day 15 (n=3-5). **(F)** Percentage and absolute number of S0 to S3 iNKT cells in control and Eed cKO fetal thymus after day 11 of FTOC culture (n=3-5). **(G)** Scheme for the generation of BM chimeras. **(H)** Percentage of TCRβ^+^CD1d-Tet^+^ iNKT cells in CD45.1^-^CD45.2^+^(Ly5.2) and CD45.1^+^CD45.2^+^ (Ly5.1/5.2) cells in the indicated organs from BM chimeras (n=5-6). Data are mean ± SD with statistical significance determined by unpaired t-test **(B, E, H)** or multiple t-tests **(D, F)**. *p* values are represented as *, <0.05; **, <0.01; ***, <0.001; ****, <0.0001. Data are representative of at least two independent experiments.

The differentiation of iNKT cells in mice begins after birth (32). To investigate potential age-related differences in iNKT cell development, we examined iNKT cell development in neonatal mice. TCRβ^+^CD1d-Tet^+^ iNKT cells were first detected at postnatal day 4 (P4), which was confirmed by the detection of NK1.1^+^ iNKT cells in P4 control mice (**Figure 2A**; **Supplementary Figure 3**). While the total numbers of thymocytes increased with postnatal days in both control and Eed cKO mice, a marked reduction in the iNKT cell fraction was observed in the Eed cKO thymus at P8 (**Figure 2B**). In control mice, the majority of iNKT cells were S2 cells; however, in Eed cKO mice, most iNKT cells were identified as S0 cells, with few S1, S2, and S3 cells (**Figures 2C, D**). We further utilized fetal thymic organ cultures to analyze iNKT cell development from embryonic progenitors. In FTOC, progenitors from the E15.5 fetal thymic lobe revealed a significant reduction in Eed-deficient iNKT cell numbers after day 11 of culture compared to that of controls, primarily due to a severe reduction in S2/3 cells, consistent with the neonatal results (**Figures 2E, F**).

CD1d and SLAM family receptors play critical roles in initial iNKT cell development (11, 33). To assess whether Eed affects the expression of these molecules, we measured the expression of CD1d, CD150 (Slamf1), and Ly108 (Slamf6) in DP thymocytes, and found no obvious differences between control and Eed cKO mice (**Supplementary Figure 4A**). In mice, over 80% of CD1d-restricted TCRαβ^+^ iNKT cells express Vα14-Jα18 chain combined with Vβ8, Vβ7, or Vβ2 chain (7). Therefore, we determined TCR Vβ usage on thymic iNKT cells in Eed cKO mice, finding no abnormalities in the distribution of TCRVβ2, Vβ5, Vβ7, Vβ8.1/8.2, and Vβ8.3 (**Supplementary Figures 4B, C**). Additionally, mRNA expression level of the Vα14-Jα18 chain was similar between control and Eed cKO DP cells (**Supplementary Figure 4D**). Thus, the abnormal development of iNKT cells in Eed cKO mice was not due to dysregulated expression of CD1d, SLAM family, Vα14-Jα18 chain, or Vβ usage on DP thymocytes.

Considering that the cellular interaction between DP thymocytes in the thymic microenvironment is crucial for the initial development of iNKT cells (4, 34), we aimed to discriminate the environmental influences caused by Eed deletion in other cell types from cell-intrinsic defects in iNKT cells. Therefore, we isolated BM cells from Ly5.2 control and Eed cKO mice and transferred them together with Ly5.1/5.2 wild-type BM cells into congenic Ly5.1 mice. The results showed a significant reduction in the percentage of iNKT cells developed from Eed cKO BM cells compared to that from control BM cells (**Figures 2G, H**). In summary, Eed is critically required for iNKT progenitors to differentiate into mature iNKT cells in a cell-intrinsic manner, particularly at the transition from the S0/S1 to S2 stage in embryos/neonates and from S2 to S3 in adults.

### 3.3 Eed is indispensable for NKT1 and NKT17 cell development

**Figure 3:**
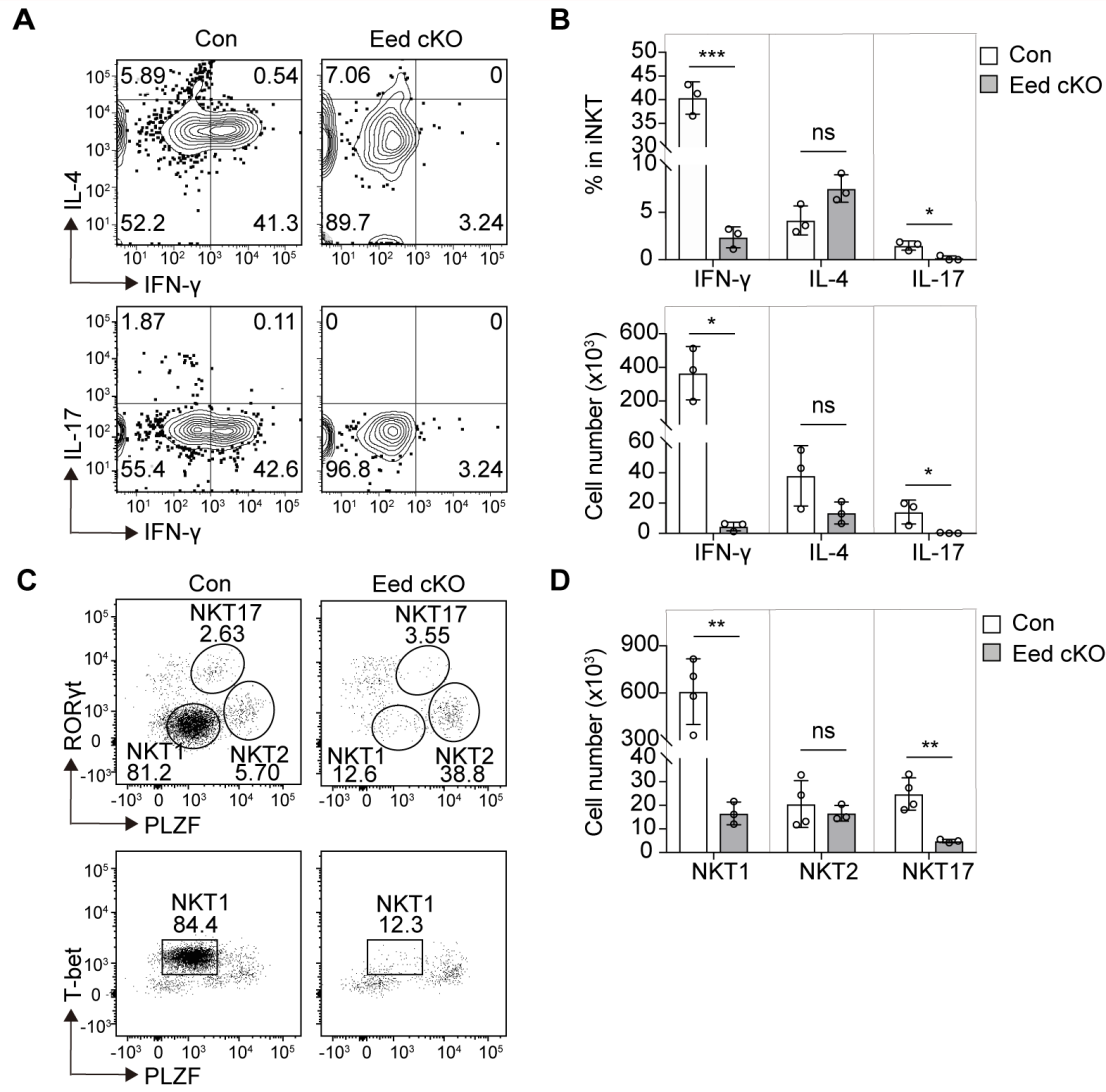
Eed is indispensable for the development of NKT1 and NKT17 cells. **(A, B)** Representative FACS plots, percentage, and absolute number of thymic TCRβ^+^CD1d-Tet^+^ iNKT cells with IFN-γ, IL-4, or IL-17 expression from control and Eed cKO mice (n=3 each). **(C, D)** Representative FACS plots and absolute number of thymic TCRβ^+^CD1d-Tet^+^ iNKT cells with T-bet, PLZF, and/or RORγt expression, determined as NKT1 (T-bet^+^PLZF^low^), NKT2(RoRγt^-^PLZF^hi^) and NKT17 (RORγt^+^PLZF^med^) from control (n=4) and Eed cKO (n=3) mice. Data are mean ± SD with statistical significance determined by unpaired t-test. *p* values are represented as *, <0.05; **, <0.01; ***, <0.001. ns, not significant. Data are representative of at least two independent experiments.

Shortly after the initial PLZF expression, iNKT cells transition to a progenitor state known as NKTp, which further differentiates into three mature NKT subsets exhibiting distinct effector functions and cytokine secretion (16). We examined the effect on the terminal maturation of iNKT subsets in Eed cKO mice based on the cytokine profiles of thymic iNKT cells, observing a significant reduction in both the percentage and cell number of IFN-γ-producing NKT1 cells and IL-17-producing NKT17 cells in Eed cKO mice compared to that of control mice, while those of IL-4-producing NKT2 cells were comparable with control mice (**Figures 3A, B**).

Different NKT subsets are characterized by specific transcription factors, including NKT1 (RORγt^lo^PLZF^lo^T-bet^+^), NKT2 (RORγt^lo^PLZF^hi^), and NKT17 (RORγt^hi^PLZF^int^) (16). Further analysis based on transcription factor expression revealed a significant reduction in NKT1 and NKT17 cells in Eed cKO mice, whereas NKT2 cells remained comparable to those in the control mice (**Figures 3C, D**). Moreover, distinct iNKT subsets defined by cell-surface markers demonstrated that CD122^+^ NKT1 and CD138^+^ NKT17 cells were significantly decreased in Eed cKO mice, whereas the absolute number of CCR7^-^PD-1^+^ NKT2 cells in Eed cKO mice was comparable to that in control mice (**Supplementary Figure 5**). Since we detected Eed expression in NKT1, NKT2, and NKT17 cells, a different requirement of Eed in NKT subsets is not due to different expression levels of Eed (**Supplementary Figure 6**). Overall, our findings suggest that Eed is indispensable for the development of both NKT1 and NKT17 cells, but not NKT2 cells.

### 3.4 Eed regulates H3K27me3 markers and iNKT cell transcriptional program

**Figure 4:**
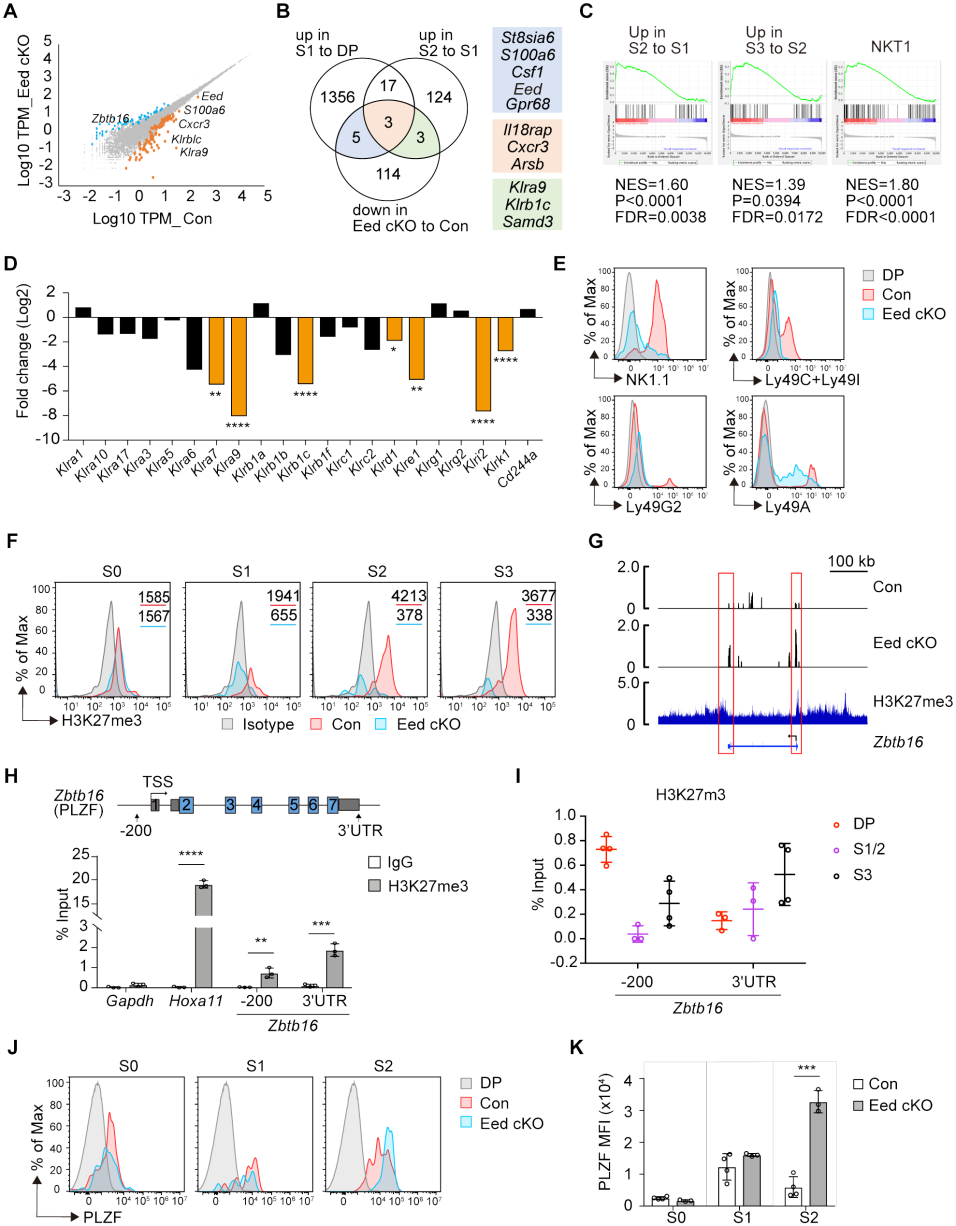
Eed is responsible for H3K27me3 marks and transcriptional programming of iNKT cells. **(A)** RNA-seq analysis on CD4^+^CD8^+^TCRβ^+^CD69^+^ thymocytes from control and Eed cKO mice (n=3 each). The scatter plot shows genes that were differentially expressed with p<0.05 and fold change >2 (*orange*) or <0.5 (*blue*) in control compared to Eed cKO mice. **(B)** Venn diagram of downregulated DEGs in Eed cKO mice to control with the gene datasets from the publicly available database (29) that upregulated in S1 to DP or S2 to S1. The number of genes is indicated in each compartment. **(C)** DEGs from control to Eed cKO DP cells are used for GSEA analysis with gene sets obtained from publicly available databases (28). Upregulated in S2 to S1 iNKT cells (*left*), upregulated in S3 to S2 iNKT cells (*middle*), and NKT1 cells (*right*). **(D)** The relative expression of NKRs in DP thymocytes. Fold changes of mRNA expression in Eed cKO mice to control mice are shown. **(E)** Surface expression of NK1.1(*Klrb1c*), Ly49C/Ly49I (*Klra3/Klra7*), Ly49G2 (*Klra9*), and Ly49A(*Klra1*) on S2/3 (CD24^-^CD44^+^) iNKT cells from control (*red*) and Eed cKO (*blue*) mice, with DP cells from control mice (*gray*). **(F)** H3K27me3 level in the indicated thymic iNKT cells from control (*red*) and Eed cKO (*blue*) mice. The gray histogram indicates isotype control. The values in each panel show the MFI. **(G)** H3K27me3 ChIP-seq peaks at the *Zbtb16* locus in iNKT cells (*bottom*) are shown with RNA-seq tracks in control (*top*) and Eed cKO (*middle*) DP cells. Representative data from triplicated pairs of control and Eed cKO mice. H3K27me3 ChIP-seq dataset is from GSE84238. **(H)** ChIP-qPCR analysis of H3K27me3 (*filled bars*) and control IgG (*open bars*) enrichments at the proximal promoter and 3’UTR region of *Zbtb16* locus in sorted control thymic iNKT cells (n=3). *Gapdh* and *Hoxa11* were used as the negative and positive controls, respectively. **(I)** ChIP-qPCR analysis of H3K27me3 enrichment at the *Zbtb16* locus in DP, S1/2, and S3 cells (n=3-4). **(J, K)** Representative FACS histograms and the MFI of PLZF in the indicated iNKT cells from control (*red*, n=4) and Eed cKO (*blue*, n=3) mice. The gray histogram indicates DP cells from control mice. Data are mean ± SD with statistical significance determined by unpaired t-test **(D, H)** or multiple t-tests **(K)**. *p* values are represented as *, <0.05, **, <0.01; ***, <0.001; ****, <0.0001. Data are representative of at least two independent experiments.

To understand how Eed deficiency inhibits iNKT cell development at the transcriptional level, we performed RNA-sequencing (RNA-seq) analysis on CD4^+^CD8^+^TCRβ^+^CD69^+^ thymocytes from control and Eed cKO mice. We identified 83 upregulated and 125 downregulated genes (fold change >2 or <0.5, p <0.05) in Eed cKO mice compared to the control (**Figure 4A**). These differentially expressed genes (DEGs) were compared with previously reported iNKT cell stage-specific datasets (29), revealing that 11 of the 125 downregulated genes in Eed cKO thymocytes. *St8sia6*, *S100a6*, *Csf1*, *Eed*, *Gpr68*, *Il18rap*, *Cxcr3*, and *Arsb* were downregulated during the DP to S1 transition, whereas *Il18rap*, *Cxcr3*, *Arsb*, *Klra9*, *Klrb1c*, and *Samd3* were downregulated during the S1 to S2 transition in an Eed-dependent manner, suggesting the importance of Eed in the developmental transition of iNKT cells (**Figure 4B**). Furthermore, S1 to S2, S2 to S3, and NKT1 transition datasets positively correlated with DEGs in control to Eed cKO DP cells (**Figure 4C**).

We then analyzed genes associated with natural cell function. Among the NK receptors (NKRs) normally expressed at the S2/S3 stage, the expression of *Klra7*, *Klra9*, *Klrb1c*, *Klrd1*, *Klre1*, *Klri2*, and *Klrk1* were significantly downregulated in Eed cKO cells (**Figure 4D**). We confirmed reduced protein levels of NK1.1 (*Klrb1c*), Ly49C/Ly49I (*Klra3*/*Klra7*), and Ly49G2 (*Klra9*) in S2/3 (CD44^+^CD24^-^) iNKT cells in Eed cKO mice (**Figure 4E**). These results indicate that Eed participates in the transcriptional regulation of various genes related to the effector function of iNKT cells.

We observed upregulation of *Zbtb16*, encoding the zinc-finger transcription factor PLZF, in Eed cKO DP cells (**Figure 4A**), whose expression is normally upregulated in S1/2 and then downregulated in S3 during iNKT cell differentiation (29). This suggests that the failure of iNKT cell development in Eed cKO mice may be associated with abnormal expression of PLZF due to a lack of H3K27me3-mediated inactivation of *Zbtb16*. Consequently, we first assessed the impact of Eed deletion on H3K27me3 levels in iNKT cells. Intracellular staining of H3K27me3 in thymocytes revealed a significant reduction of H3K27me3 levels in Eed cKO iNKT cells compared to that of controls at the S1, S2, and S3 stages by 3.0-, 11.1-, and 10.9-fold, respectively (**Figure 4F**). To further understand the relationship between H3K27me3 status and *Zbtb16* transcription, we compared the peaks from RNA-seq and H3K27me3 Chromatin immunoprecipitation sequencing (ChIP-seq) obtained from iNKT cells around the *Zbtb16* locus (30). H3K27me3 signals were detected at the transcriptional start and end regions of *Zbtb16*, corresponding to elevated RNA-seq peaks in Eed cKO cells (**Figure 4G**; **Supplementary Table 2**). Additionally, we detected H3K27me3 signals at the proximal promoter and 3′ untranslated region (3′ UTR) of *Zbtb16* in iNKT cells, along with a known Eed target *Hoxa11* (**Figure 4H**) (35). Notably, H3K27me3 enrichment was higher at the proximal promoter region of *Zbtb16* in the DP stage, followed by a decline in the S1/S2 stage and subsequent recovery in the S3 stage, inversely correlating with the PLZF expression pattern (**Figure 4I**). As expected, PLZF protein expression significantly increased in Eed cKO iNKT cells at the S2 stage (**Figures 4J, K**). Thus, Eed likely negatively regulates PLZF expression through H3K27me3 at the proximal promoter and 3′ UTR region of *Zbtb16* during iNKT cell development.

### 3.5 Increased cell death in Eed-deficient iNKT cells

**Figure 5:**
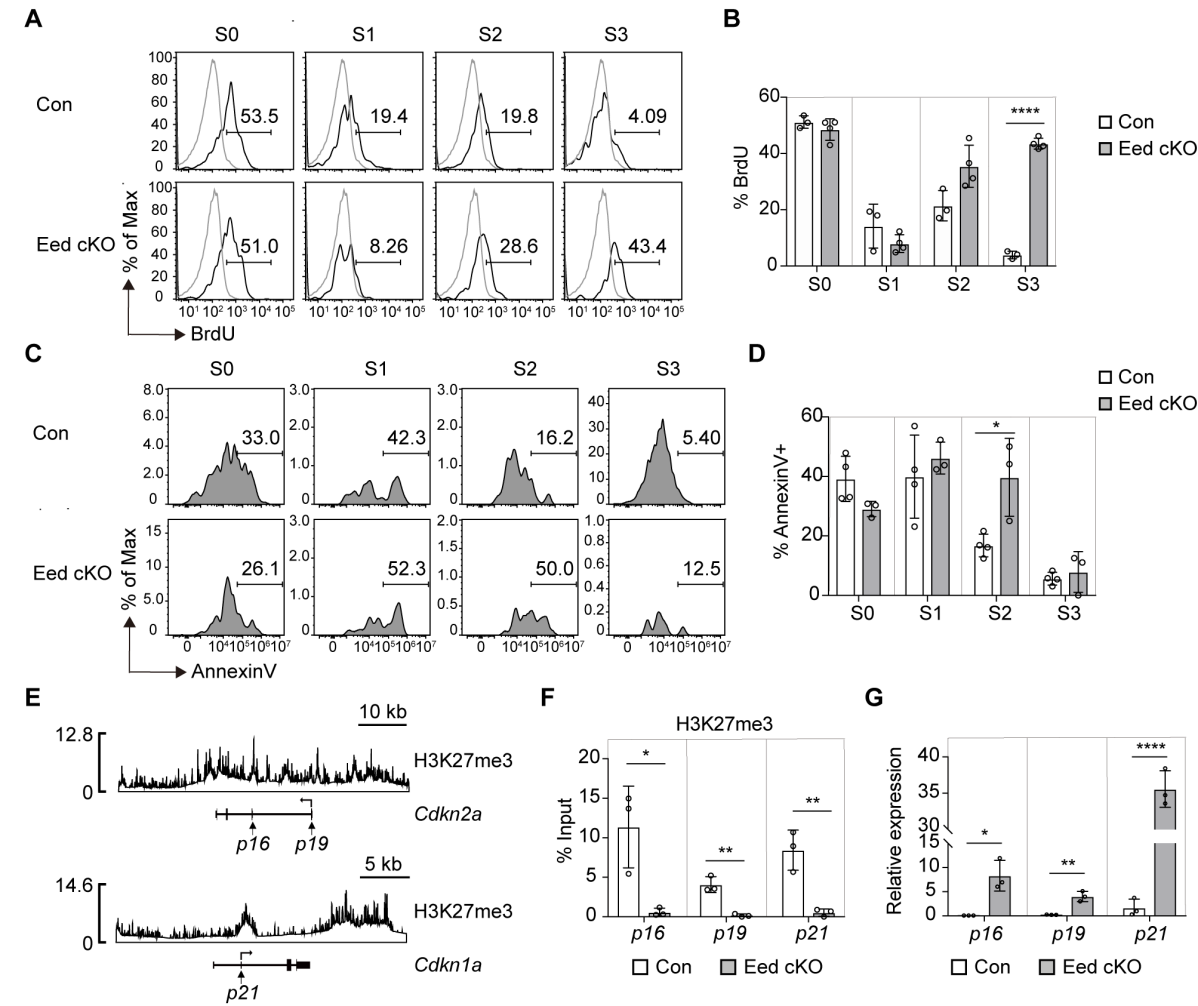
Increased cell death in Eed-deficient iNKT cells is associated with abnormal expression of *Cdkn2a* and *Cdkn1a.* **(A, B)** Representative FACS histograms and percentage of BrdU^+^ iNKT cells in the thymus from BrdU-pulsed control (n=3) and Eed cKO (n=4) mice. The gray line indicates CD4^+^CD8^-^SP cells. **(C, D)** Representative FACS histograms and percentage of Annexin V^+^-dead cells in the indicated iNKT cells from control (n=4) and Eed cKO (n=3) mice. **(E)** ChIP-seq analysis of H3K27me3 in iNKT cells at the *Cdkn2a* locus (*upper*) and *Cdkn1a* locus (*lower*). **(F)** ChIP-qPCR analysis of H3K27me3 enrichment at the proximal promoter region of *p16*, *p19*, and *p21* in sorted thymic CD24^-^NK1.1^-^ S1/2 iNKT cells from control and Eed cKO mice (n=3 each). **(G)** Real-time PCR analysis of the expression of *p16*, *p19*, and *p21* in sorted thymic S2 iNKT cells from control and Eed cKO mice (n=3 each). These expressions were normalized to *Ywhaz*. Data are mean ± SD with statistical significance determined by unpaired t-test **(F, G)** or multiple t-tests **(B, D)**. *p* values are represented as *, <0.05; **, <0.01; ****, <0.0001. Data are representative of at least two independent experiments.

The proliferation and survival of iNKT cells significantly influence their development and population size (7). We evaluated these aspects in Eed cKO iNKT cells by assessing BrdU incorporation for proliferation and Annexin V staining for survival. While BrdU incorporation rates in Eed cKO iNKT cells were similar to or higher than those in control mice, a significant increase in the percentage of Annexin V^+^ iNKT cells was observed at the S2 stage in Eed cKO mice (**Figures 5A-D**). This indicates that impaired cell survival, particularly at the S2 developmental stage, contributes to developmental defects in Eed-deficient iNKT cells.

Tumor suppressor *Cdkn2a*, a target of PRC2 (36), encodes two proteins (p16Ink4a and p19Arf) that promote cell cycle arrest and apoptosis by regulating RB and p53 proteins, respectively (37). Additionally, *Cdkn1a*, encoding a p21 CDK inhibitor, is involved in the p53-RB signaling pathway that regulates the cell cycle (38). Both *Cdkn2a* and *Cdkn1a* are implicated in apoptosis induction in hematopoietic stem cells following Eed deficiency (36). We hypothesized that the abnormal expression of *Cdkn2a* and *Cdkn1a* contributes to the impaired survival and development of Eed-deficient iNKT cells. H3K27me3 ChIP-seq analysis using iNKT cells revealed H3K27me3 signals around the transcriptional promoter and 3′ UTR region of *Cdkn2a* and *Cdkn1a* in iNKT cells (**Figure 5E**). Moreover, proximal promoter regions of *Cdkn2a* and *Cdkn1a* were significantly enriched with H3K27me3 in control S1/2 cells in an Eed-dependent manner (**Figure 5F**). Furthermore, mRNA expression of *p16Ink4a*, *p19Arf*, and *p21* was significantly increased in S2 cells from Eed cKO mice compared to that in the control (**Figure 5G**). These results strongly support that *Cdkn2a* and *Cdkn1a* are direct targets of Eed for H3K27me3, regulating cell death and promoting iNKT cell development.

### 3.6 Increased susceptibility to liver injury and inflammation in Eed cKO mice

**Figure 6:**
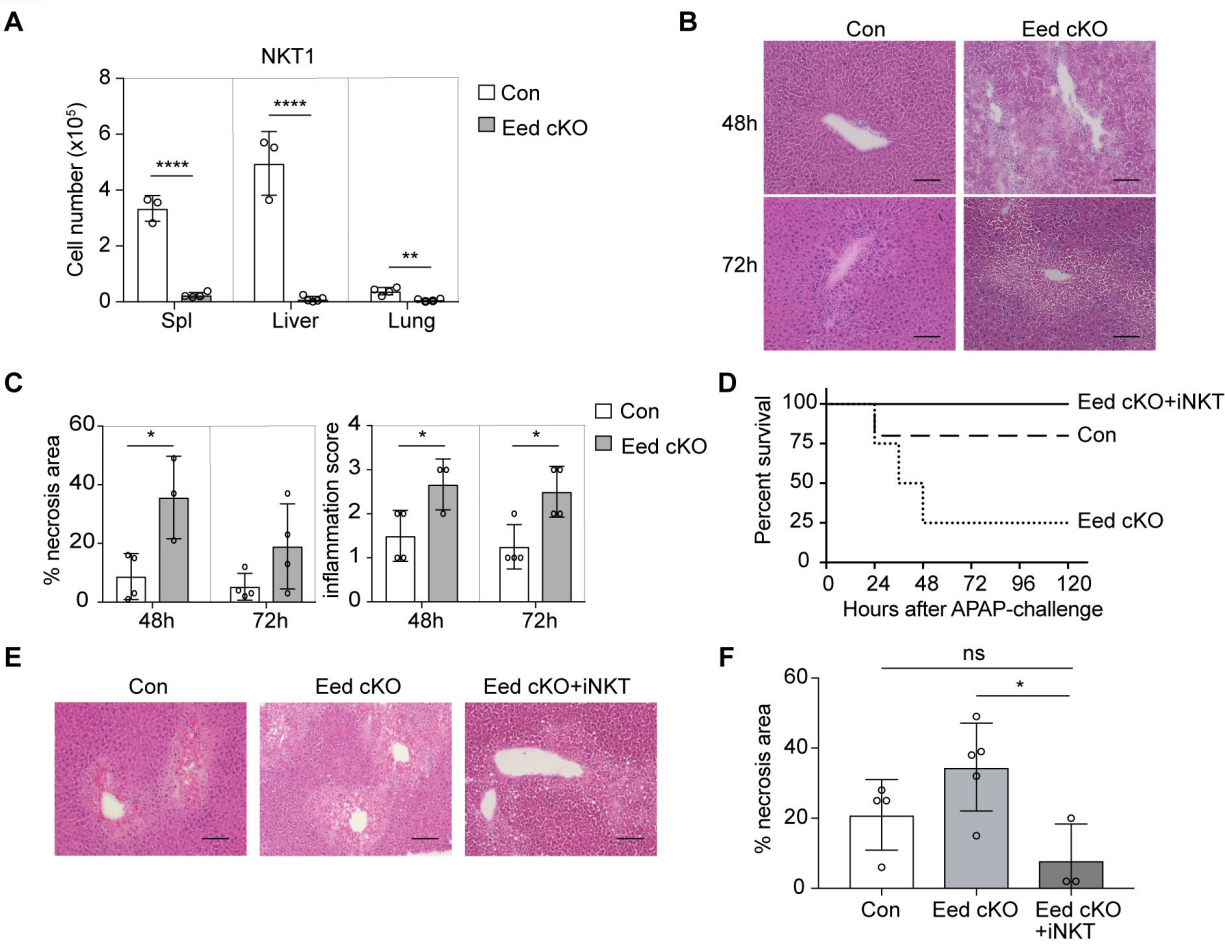
Increased susceptibility to liver injury in Eed-deficient mice. **(A)** Absolute cell number of NKT1 in the spleen, liver, and lung from control (n=3-4) and Eed cKO (n=4) mice. **(B)** Representative images of H&E staining of the liver from control and Eed cKO mice at 48 h and 72 h after APAP treatment. Image scale, 100 μm. **(C)** Percentage of necrosis area and inflammation score in the livers of B (n=3-4 each). **(D)** Survival curve after APAP treatment in control (*dotted line*), Eed cKO (*dashed line*) mice, and Eed cKO mice transferred with iNKT cells from wild-type liver (Eed cKO+iNKT, *solid line*) (n=3-5 each). **(E)** Representative images of H&E staining of the liver at 24 h after APAP treatment of the indicated mice. Image scale, 100 μm. **(F)** Percentage of necrosis area in the livers of **(E)** Data are mean ± SD with statistical significance determined by unpaired t-test **(A, C)**, or one-way ANOVA **(F)**. *p* values are represented as *, <0.05; **, <0.01; ****, <0.0001. n.s., not significant. Data are representative of at least two independent experiments.

As Eed deficiency leads to a reduction in thymic NKT1 cells, we examined NKT1 cells in peripheral tissues and observed a substantial proportion residing in the liver. However, the numbers of NKT1 cells in the spleen, liver, and lung of Eed cKO mice were significantly diminished (**Figure 6A**). CD1d- and Jα18-deficient mice lacking iNKT cells are highly susceptible to acetaminophen (APAP)-induced liver injury (AILI) (39, 40), a severe consequence of sudden hepatocyte injury, often induced by APAP overdose in developed countries (41), but the critical iNKT cell subset involved and the role of epigenetic modification remain unknown. To investigate the physiological significance of Eed-dependent iNKT cell development, we used an AILI mouse model. Compared with control mice, Eed cKO mice exhibited a lower survival rate after APAP challenge, with mortality being dose-dependent (**Supplementary Figure 7A**). Histological analysis of the livers collected 48h or 72h post-APAP treatment revealed significantly enlarged necrotic areas accompanied by severe inflammation in the liver of Eed cKO mice compared to controls (**Figures 6B, C**). In APAP-treated mice, iNKT cells were significantly reduced in Eed cKO mice, whereas the numbers of CD4^+^ and CD8^+^ T cells were comparable to those in control mice (**Supplementary Figures 7B, C**). To determine whether the defect induced in APAP-treated Eed cKO mice resulted from a lack of iNKT cells, we transferred iNKT cells from the wild-type liver (mostly NKT1 cells) into Eed cKO mice. The results showed that iNKT cell transfer significantly increased survival and reduced necrosis in Eed cKO mice 24 h after APAP treatment (**Figures 6D–F**). Collectively, Eed-dependent liver resident NKT cells, particularly NKT1 cells, play a critical role in protecting hepatocytes from APAP-mediated necrosis and liver inflammation.

## 4 Discussion

Eed plays a unique role in initiating Ezh1/2 activation and amplifying PRC2 activity through its interaction with H3K27me3, essential for H3K27me3 deposition (20, 21). Here, we demonstrated that H3K27me3 levels significantly increase with iNKT cell differentiation, and Eed is crucial for maintaining these marks and facilitating cell differentiation. While both Ezh2 and Eed are integral to PRC2, the diametrically opposite iNKT cell phenotypes between Ezh2 cKO and Eed cKO mice indicate distinct roles of these proteins in iNKT cell development (22, 23). In contrast to the significant reduction of H3K27me3 due to Eed deficiency, H3K27me3 levels were not impacted by Ezh2 deficiency in iNKT cells (23). Therefore, H3K27me3 in iNKT cells may be primarily catalyzed by the PRC2 complex consisting of Ezh1 and Eed. H3K27me3 level is higher in S2 and S3 iNKT cells, while Eed expression is higher in S1 and S2 iNKT cells. This discrepancy could be explained by the PRC2-mediated amplification of H3K27me3 to neighboring sites of the initial trimethylations. After the initial H3K27me3 deposition at S1 stage, PRC2 is recruited by binding of Eed to H3K27me3, leading to further H3K27me3 deposition in a positive feedback loop, which could cumulatively increase H3K27me3 levels in S2 and S3 iNKT cells. However, sustained H3K27me3 levels caused by the loss of the H3K27me3 demethylases Utx and Jmjd3 also affect iNKT cell development. UTX-deficient iNKT cells exhibit impaired expression of iNKT-cell signature genes due to decreased activation-associated H3K4me3 markers and increased repressive H3K27me3 markers within the promoters occupied by UTX (30). Utx- or Jmjd3-deficient iNKT cells fail to activate PLZF and its target genes, resulting in reduced iNKT cells (22, 30). Therefore, the Eed-PRC2 histone methyltransferase and Utx/Jmjd3 histone demethylases may differentially regulate H3K27me3 target genes, leading to similar knockout phenotypes.

PLZF, exclusively expressed in iNKT cells, is induced immediately after positive selection of iNKT cell precursors. During iNKT cell development, PLZF expression reaches a plateau at the S1 stage and then declines markedly at the S3 stage (14). In PLZF-deficient mice, iNKT cells showed impaired intra-thymic expansion accompanied by a significant reduction in the thymus, spleen, and liver, lacked NK markers and failed to secrete effector cytokines after activation (14, 15). Therefore, properly organized PLZF expression directs innate-like effector differentiation from T-cell progenitors. Our data indicated that Eed-mediated upregulation of H3K27me3 at S2/3 silences the *Zbtb16* locus, promoting NKT1 and NKT17 differentiation. This model is supported by observations in PLZF-active mutant mice, where constitutively active PLZF inhibits the progression of NKT precursors to subsequent differentiation into NKT1 and NKT17, but not NKT2 cells (42). Moreover, the absence of the PRC2 components, Ezh2 or Jarid2, upregulates the PLZF protein by modulating the stability of PLZF without altering H3K27me3, thereby expanding iNKT cells with the IL-4 secreting NKT2 phenotype, indicating the significance of PLZF downregulation at the S2 stage (23, 43). Eed deletion resulted in an elevation of the PLZF protein at S2, as observed in Ezh2- or Jarid2-deficient mice; however, there was a significant reduction in thymic and peripheral iNKT cells without NKT2 expansion, suggesting additional roles of Eed.

iNKT cells migrate to the peripheral tissues after development; however, the physiological significance of Eed-dependent iNKT cells remains unclear. Here, we demonstrated that T cell-specific Eed-deficient mice are highly susceptible to AILI and associated inflammation due to a lack of iNKT cells. In AILI, metabolized APAP forms cytotoxic mitochondrial adducts that cause hepatocyte death and the release of danger-associated molecules, thereby promoting the release of pro-inflammatory cytokines (44). Although how iNKT cells protect hepatocytes from death remains unclear, liver-resident iNKT cells may provide anti-apoptotic signals to hepatocytes through effector molecules like IFN-γ and IL-17 or CD1d, which is constitutively expressed on hepatocytes (45, 46).

Eed-deficient iNKT cells show increased induction of senescence-associated genes such as *p16Ink4a*, *p19Arf*, and *p21*, promoting apoptosis, cell cycle arrest, senescence, and inflammation (47). This enhanced apoptosis could be attributed to the induction of p19Arf expression via p53-dependent or -independent pathways (48). However, CD8^+^ T cells can eliminate p16Ink4a-positive senescent cells, which ameliorates various age-related mouse phenotypes (49). Therefore, the enhanced apoptosis observed in Eed-deficient iNKT cells may be the result of cytotoxic CD8^+^ T cells targeting senescent iNKT cells.

NKT1 and NKT17 cells differentiate from NKTp cells at different rates, with NKT17 cells emerging within 3–5 days and NKT1 cells requiring 14–20 days, contrasting with NKT2 cells remaining PLZF^hi^ similar to NKTp (50). Thus, NKT1 development involves more complex processes, including the IL-15 pathway and T-bet-mediated regulation. Our findings shed light on the role of Eed-mediated epigenetic modifications in determining iNKT cell expansion and differentiation through the orchestration of iNKT cell-specific transcriptional programs.

## Data Availability

The datasets presented in this study can be found in online repositories. The names of the repository/repositories and accession number(s) can be found below: PRJNA1054693 (SRA).
