# Supplementary material for: Eed-dependent histone modification orchestrates the iNKT cell developmental program alleviating liver injury
Source: Front Immunol. 2024 Sep 20;15:1467774. doi: 10.3389/fimmu.2024.1467774 (PMC11449725; doi:10.3389/fimmu.2024.1467774)
Supplement: Supplementary file 1 [file DataSheet1.pdf]

Supplementary Material

1 Supplementary Data

Supplementary Material includes 7 Supplementary Figures and 2 Tables.

2 Supplementary Figures and Tables

2.1 Supplementary Figures

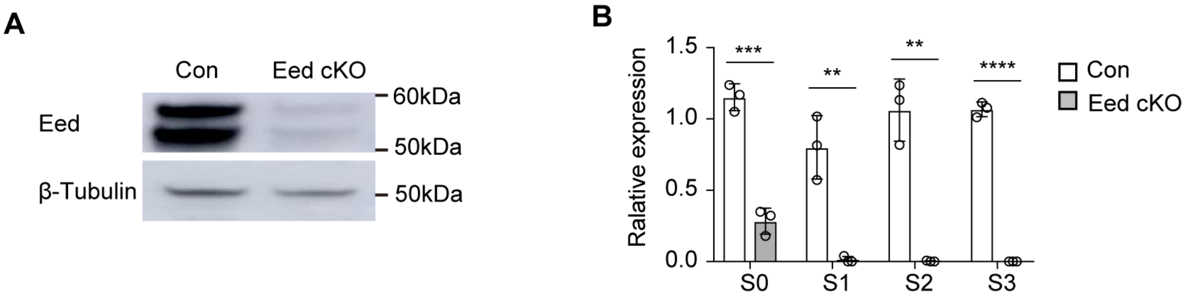

Supplementary Figure 1. The expression of Eed in splenic T cells and thymic iNKT cells

(A) Western blotting analysis of Eed expression was performed on purified splenic T cells from control and Eed cKO mice. β-Tubulin was detected as a loading control. (B) Real-time PCR analysis of *Eed* expression in the indicated thymic iNKT cell populations from control and Eed cKO mice (n=3 each). S0, $CD24^+$; S1, $CD24^-CD44^-NK1.1^-$; S2, $CD24^-CD44^+NK1.1^-$; and S3, $CD24^-CD44^+NK1.1^+$ cells are gated on $TCR\beta^+CD1d\text{-}Tet^+$iNKT cells. The expressions were normalized to *Ywhaz*. Data are Mean ± SD with statistical significance determined by multiple t-tests. *p* values are represented as **, <0.01; ***, <0.001; ****, <0.0001. Data are representative of two independent experiments.

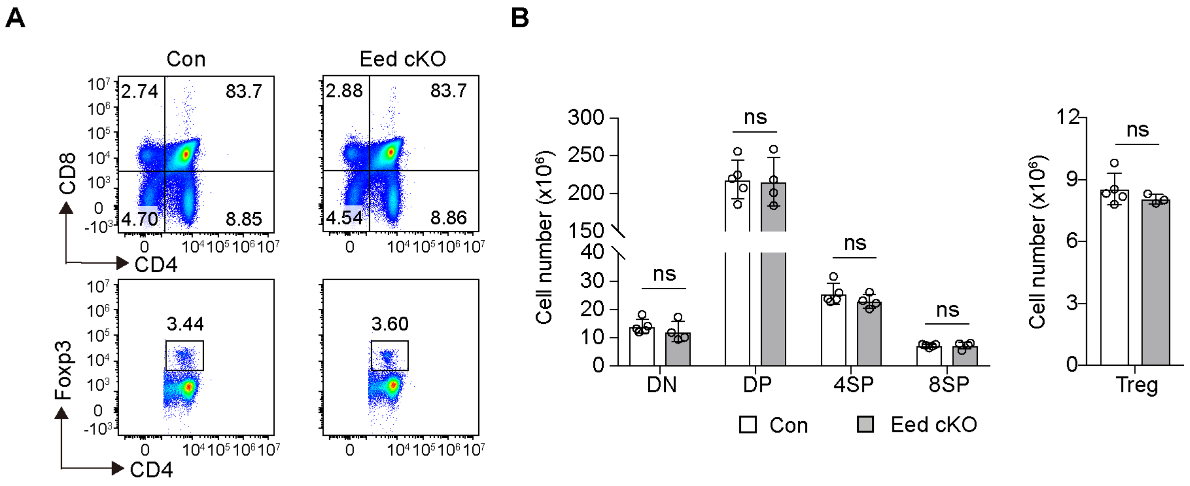

Supplementary Figure 2. Eed is dispensable for the development of thymocyte and thymic regulatory T cells

(A) Representative FACS plot of CD4, CD8, and Foxp3 expression in the thymus from control and Eed cKO mice. (B) Absolute cell number of the indicated subsets in the thymus from control (n=5) and Eed cKO (n=4) mice. DN, $CD4^{-}CD8^{-}$; DP, $CD4^{+}CD8^{+}$; 4SP, $CD4^{+}CD8^{-}$; and 8SP, $CD4^{-}CD8^{+}$; Treg, $CD4^{+}CD8^{-}Foxp3^{+}$. Data are mean ± SD with statistical significance determined by unpaired or multiple t-tests. ns, not significant. Data are representative of more than two independent experiments.

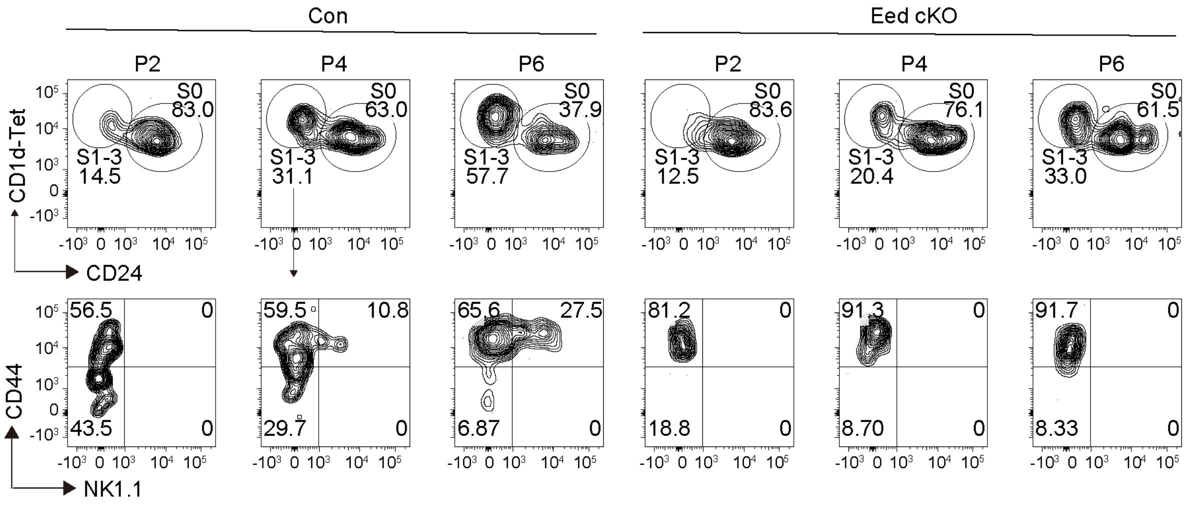

Supplementary Figure 3. The development of iNKT cells in neonatal mice.

Representative FACS plots of the thymic iNKT populations from control and Eed cKO mice at postnatal day 2 (P2), 4 (P4), and 6 (P6). Expressions of CD24, CD44, and NK1.1 are analyzed on TCRβ$^+$CD1d-Tet$^+$iNKT cells corresponding to the S0 to S3 stages. Data are representative of at least two independent experiments.

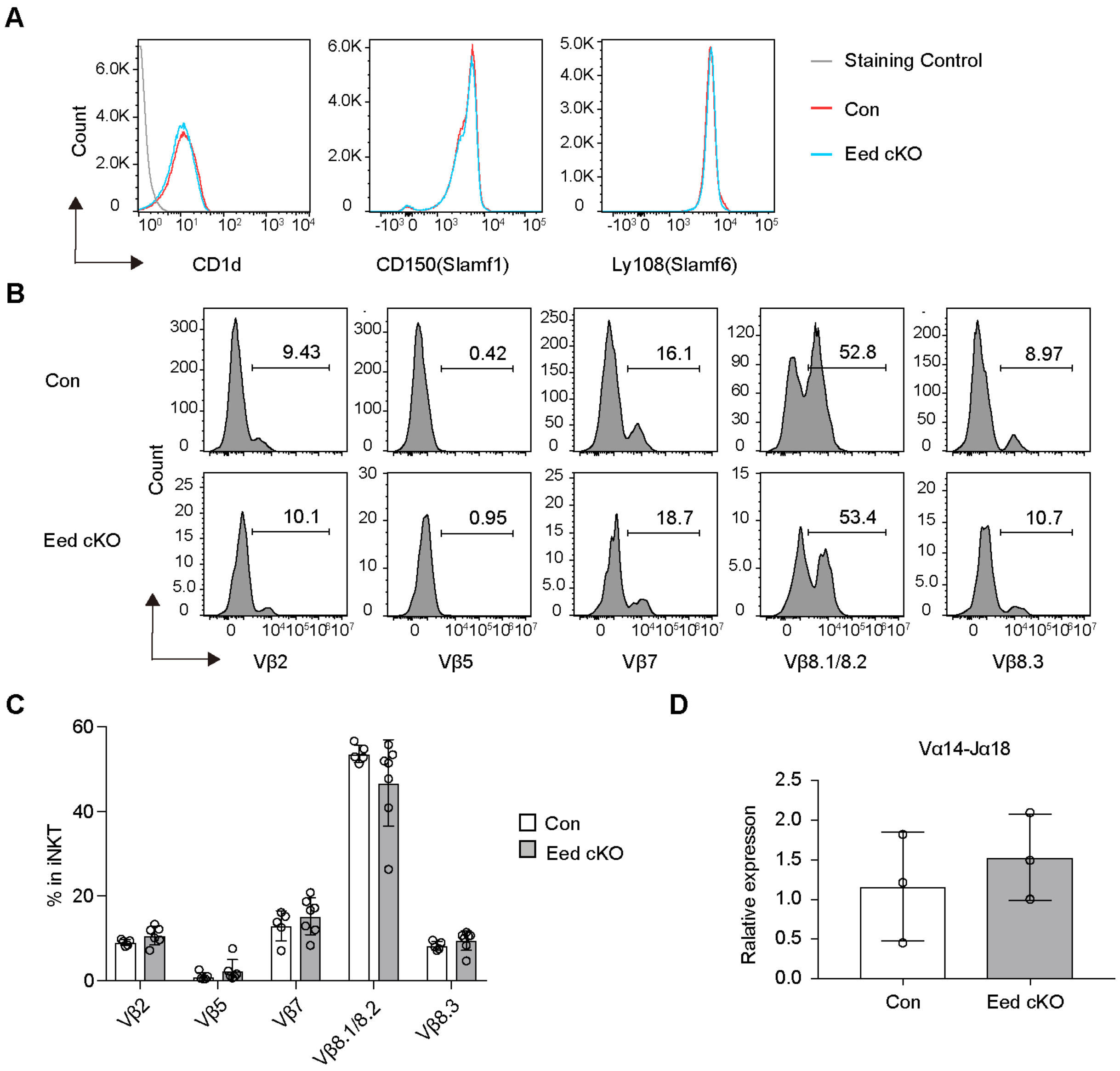

Supplementary Figure 4. The defects in Eed-deficient iNKT are independent of antigen presentation and TCRαβ rearrangement

(A) FACS analysis of CD1d, CD150, and Ly108 expression on $CD4^{+}CD8^{+}$DP thymocytes from control and Eed cKO mice. (B-C) FACS analysis and percentage of TCRVβ2, Vβ5, Vβ7, Vβ8.1/8.2, and Vβ8.3 levels in thymic iNKT cells from control (n=5) and Eed cKO (n=7) mice. (D) Real-time PCR of TCRVα14-Jα18 expression normalized to *HPRT* in DP thymocytes from control or Eed cKO mice (n=3 each). Data are mean ± SD with statistical significance determined by unpaired t-test. Data are representative of at least two independent experiments.

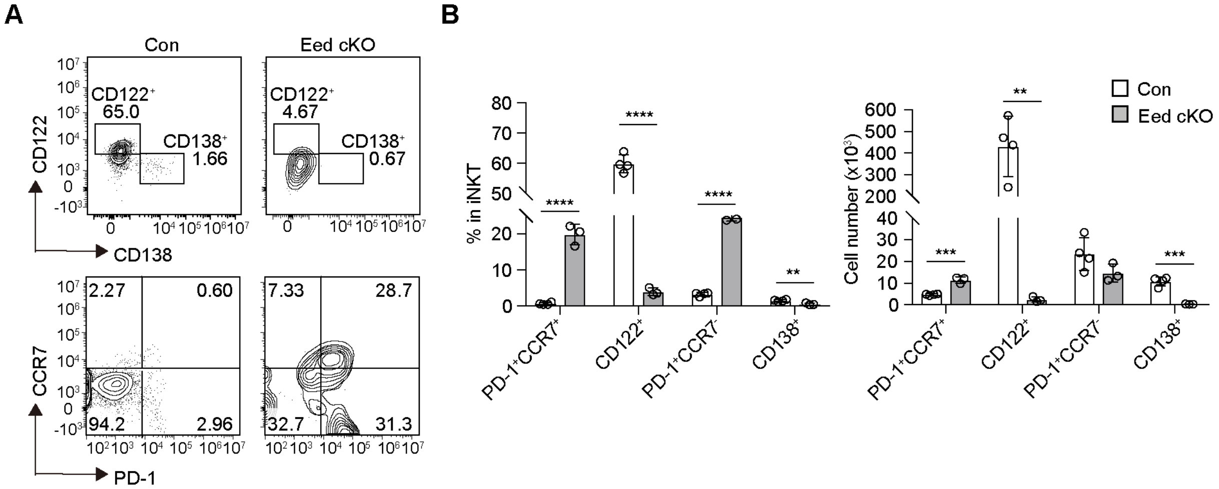

Supplementary Figure 5. Eed is essential for the development of NKT1 and NKT17 cells

(A-B) Representative FACS plot, percentage, and absolute cell number of the indicated thymic TCRβ^+CD1d-Tet$^+$CD24$^-$ iNKT populations from control and Eed cKO mice based on CD122, CD138, CCR7 and PD-1 expression and determined as CD122$^+$NKT1, CD138$^+$NKT17 and PD-1$^+$CCR7$^+$ NKTp and PD-1$^+$CCR7$^-$NKT2 cells (n=3-4 each). Data are mean ± SD with statistical significance determined by unpaired t-test. *p* values are represented as **, <0.01; ***, <0.001; ****, <0.0001. Data are representative of two independent experiments.

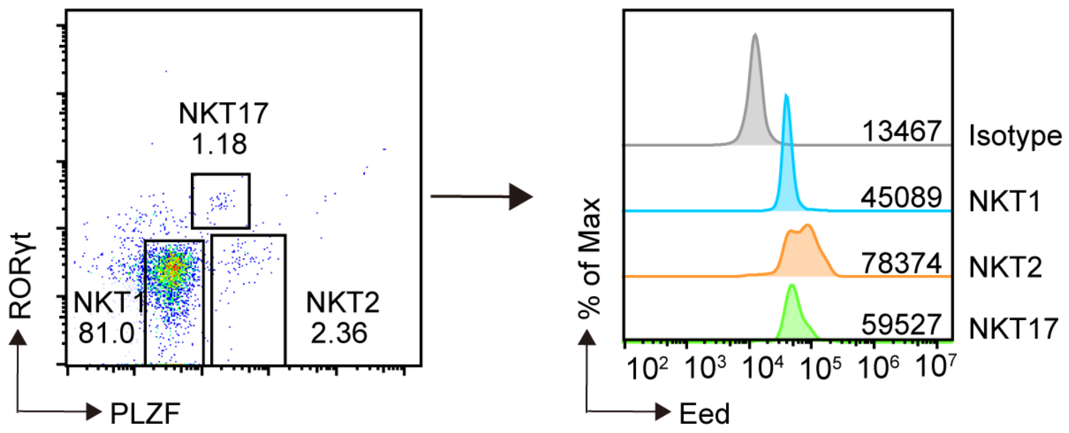

Supplementary Figure 6. The expression of Eed in iNKT cell subsets

Representative FACS plot of NKT1, NKT2, and NKT17 gated on thymic TCRβ^{+}CD1d-Tet^{+} iNKT populations from control mice based on RORγt and PLZF expression (*left)*. Eed expression was shown in histograms and the number showed MFI of each subset (*right*). Data are representative of two independent experiments.

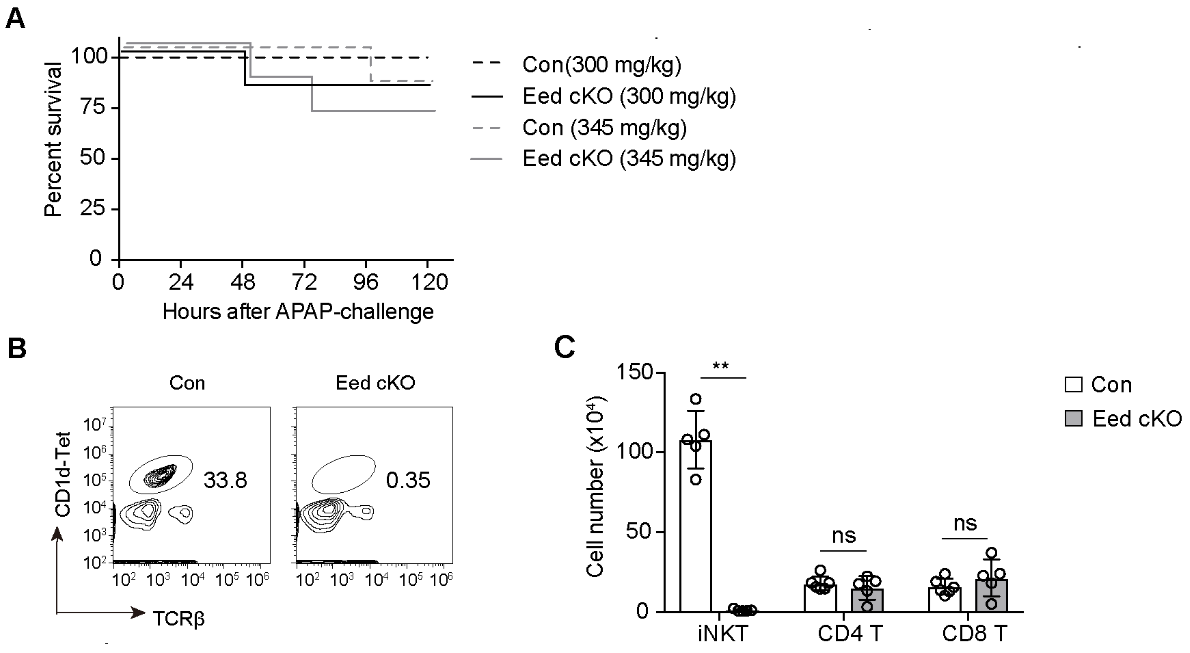

Supplementary Figure 7. APAP-induced liver injury in Eed-deficient mice

(A) Survival curve of Control and Eed cKO male mice treated with 300 mg/kg or 345 mg/kg APAP (n=3-6 each). (B) Representative FACS plot of liver iNKT cells. (C) Absolute number of iNKT cells, CD4 T ($CD4^{+}$CD1d-Tet^{-} $TCR\beta^{hi}$) cells, and CD8 T ($CD8^{+}TCR\beta^{hi}$) cells in the liver from control and Eed cKO mice at 24 h after APAP treatment (n=5 each). Data are mean ± SD with statistical significance determined by unpaired t-test. *p* values are represented as **, <0.01. ns, not significant. Data are representative of at least two independent experiments.

2.2 Supplementary Tables

Supplementary Table 1. ChIP-qPCR primers

Gene	Forward (5'-3')	Reverse (5'-3')
Hoxa11 (1)	GCTGCGAAGAAGGTGCTGAACG	CGGTGGGTGAGGGATACTCTCTGG
Gapdh (2)	GCGCGAAAGTAAAGAAAGAAGCCC	AGCGGCCCGGAGTCTTAAGTATTAG
Zbtb16 -200 (2)	CGGTGGTGATTTGCTAACCT	GAACTGTTGCTCCGGATCTC
Zbtb16 3' UTR	CGTCTGGATAGTCACGCCTC	GACCAGTGAGCAGCTTGGAT
p16Ink4a	ACACGTGTGCACTTCTTTGC	AGCGCTAAAGGATCGGACAG
p19Arf	CTCACCTCGCTTGTCACAGT	GGATCGCACGAACTTCACCA
p21	CCGACGCTATAAGGAGGCAG	CCACGAAGCTCTCACCTCTG

Reference

1. Sun B, Chang E, Gerhartl A, Szele FG. Polycomb Protein Eed Is Required for Neurogenesis and Cortical Injury Activation in the Subventricular Zone. *Cereb Cortex* (2018) 28(4):1369-82. Epub 2018/02/08. doi: 10.1093/cercor/bhx289.
2. Pereira RM, Martinez GJ, Engel I, Cruz-Guilloty F, Barboza BA, Tsagaratou A, et al. Jarid2 Is Induced by Tcr Signalling and Controls Inkt Cell Maturation. *Nat Commun* (2014) 5:4540. Epub 2014/08/12. doi: 10.1038/ncomms5540.

Supplementary Table 2. The list of DEGs from RNA-seq and the H3K27me3 ChIP-seq analyses.

FC>2 in RNA-seq & up in H3K27me3 ChIP-seq	FC<0.5 in RNA-seq & up in H3K27me3 ChIP-seq	FC>2 in RNA-seq & down in H3K27me3 ChIP-seq	FC<0.5 in RNA-seq & down in H3K27me3 ChIP-seq
Adam23	4921507P07Rik	Adcy1	Ankrd35
Arhgap42	Alpk2	Amot	Apobr
Arhgef12	Ankmy1	Apbb1	Arhgap28
Arhgef40	Apol10b	Cd163l1	Arrdc4
Asb4	BC034090	Cd209a	Arsb
Cfap126	C2cd2	Cds1	Btc
Chst11	Card10	Crip2	Cacna1h
Cldn1	Cd207	Diras2	Ccl6
Col6a1	Cib2	Echdc2	Chrnb2
Fer1l6	Ctla4	Eml1	Csf1
Gnaz	Dmxl2	Eml2	Csf1r
Gpr19	Dnah7a	Fam174b	Ctla2b
Kitl	Dyrk3	Fbxo48	Cxcr3
Map2	Eepd1	Fsd1l	D630039A03Rik
Mfsd9	Exph5	Gapt	Dnah11
Mst1r	Fn1	Gm14548	Dnase1l2
Pcdhb16	Hecw2	Gm31255	Eed
Phldb1	Il18rap	Gm44504	Ehbp1
Pkp1	Il5ra	Gm51877	Elovl2
Prss50	Klra7	Gm6710	Fam151b
Rftn2	Klra9	Gpr52	Fes
Ripk4	Klrb1c	H2ac13	Galnt3
Scara3	Klrd1	Hey1	Gas2
Tmod2	Klre1	Homer2	Gm5454
Zbtb16	Klri2	Hspb6	Gpr141
	Klrk1	Iqcd	Gpr68
	Nuak2	Kbtbd13	Gucy1a1
	Plpp2	Kcnk6	Gucy2e
	Plxna4	Krt18	H2-M2
	Ppp2r3a	Macc1	H2bc8
	Sall3	Mpp2	Hdhd3
	Samd3	Myh10	Hopx
	Serpinb12	Myo1d	Il12b
	Socs2	Myt1l	Islr
	Tbc1d8	P3h1	Itgam
	Tent5a	Pde6g	Kif24
	Tmbim1	Plat	Klf8
	Ttc12	Plch1	LOC118568006
	Zc3h12c	Pltp	LOC118568020
		Ptchd4	Lhfp
		Rapgef1l	Lhx2

		Rsad2<br>Rxfp1<br>Slc16a2<br>Slc22a23<br>Slc26a1<br>Tex11<br>Tgfb3<br>Tmem158<br>Tmem231<br>Tmod4<br>Trim16<br>Ttc39a<br>Vcan<br>Zfp108<br>Zfp947<br>Zgrf1<br>Zscan10	Loxl4<br>Lpcat2<br>Map3k9<br>Map6<br>Mcf2l<br>Mertk<br>Metrnl<br>Mpo<br>Myo19<br>Naip2<br>Naip6<br>Nbea<br>Niban2<br>Notch3<br>Nqo1<br>Osgin1<br>Otud7b<br>Pde10a<br>Pfkfb1<br>Pkn3<br>Ppp1r26<br>Prkaa2<br>Ptpn21<br>Pygl<br>Rapgef5<br>Reln<br>Rgs1<br>S100a6<br>Serpina3g<br>Shc4<br>Slc4a11<br>Spata7<br>St8sia6<br>Syt3<br>Tifab<br>Tlr13<br>Tshz2<br>Usp35<br>Vcam1<br>Vipr2<br>Wdfy3<br>Wdr60<br>Zbtb32<br>Zfp870<br>Zfp937
